# Prolonged Periods of Social Isolation From Weaning Reduce the Anti-inflammatory Cytokine IL-10 in Blood and Brain

**DOI:** 10.3389/fnins.2018.01011

**Published:** 2019-01-11

**Authors:** Fabiana Corsi-Zuelli, Helene Aparecida Fachim, Camila Marcelino Loureiro, Rosana Shuhama, Giuliana Bertozi, Sâmia Regiane Lourenço Joca, Paulo Rossi Menezes, Paulo Louzada-Junior, Cristina Marta Del-Ben

**Affiliations:** ^1^Division of Psychiatry, Department of Neuroscience and Behavior, Ribeirão Preto Medical School, University of São Paulo, Ribeirão Preto, Brazil; ^2^Biomolecular Sciences Research Centre, Sheffield Hallam University, Sheffield, United Kingdom; ^3^Division of Clinical Immunology, Department of Internal Medicine, Ribeirão Preto Medical School, University of São Paulo, Ribeirão Preto, Brazil; ^4^Department of Pharmacology, Ribeirão Preto Medical School, University of São Paulo, Ribeirão Preto, Brazil; ^5^Department of Physics and Chemistry, School of Pharmaceutical Sciences, University of São Paulo, Ribeirão Preto, Brazil; ^6^Department of Clinical Medicine, Translational Neuropsychiatry Unit, Aarhus University, Aarhus, Denmark; ^7^Department of Preventive Medicine, Faculdade de Medicina, Universidade de São Paulo, São Paulo, Brazil

**Keywords:** anti-inflammatory cytokines, early stress, cytokines, inflammation, interleukin-10, post-weaning social isolation, schizophrenia, social isolation rearing

## Abstract

Life stressors during critical periods are reported to trigger an immune dysfunction characterised by abnormal production of inflammatory cytokines. Despite the relationship between early stressors and schizophrenia is described, the evidence on inflammatory biomarkers remains limited. We aimed to investigate whether an imbalance between pro- and anti-inflammatory cytokines in the brain is reflected in the peripheral blood of rats submitted to post-weaning social isolation (pwSI), a model with validity to study schizophrenia. We evaluated pro- and anti-inflammatory cytokines (IL-6, TNF-α, and IL-10) simultaneously at blood, prefrontal cortex and hippocampal tissues (Milliplex MAP), including the respective cytokines gene expression (mRNA) (qRT-PCR TaqMan mastermix). We also performed a correlation matrix to explore significant correlations among cytokines (protein and mRNA) in blood and brain, as well as cytokines and total number of square crossings in the open field for isolated-reared animals. Male *Wistar* rats (*n* = 10/group) were kept isolated (*n* = 1/cage) or grouped (*n* = 3–4/cage) since weaning for 10 weeks. After this period, rats were assessed for locomotion and sacrificed for blood and brain cytokines measurements. Prolonged pwSI decreased IL-10 protein and mRNA in the blood, and IL-10 protein in the hippocampus, along with decreased IL-6 and its mRNA expression in the prefrontal cortex. Our results also showed that cytokines tend to correlate to one-another among the compartments investigated, although blood and brain correlations are far from perfect. IL-10 hippocampal levels were negatively correlated with hyperlocomotion in the open field. Despite the unexpected decrease in IL-6 and unchanged TNF-α levels contrast to the expected pro-inflammatory phenotype, this may suggest that reduced anti-inflammatory signalling may be critical for eliciting abnormal behaviour in adulthood. Altogether, these results suggest that prolonged early-life adverse events reduce the ability to build proper anti-inflammatory cytokine that is translated from blood-to-brain.

## Introduction

Rearing rat pups in isolation since weaning significantly interferes with the morphological and neurochemical development of the early postnatal brain, contributing to long-term maladaptive behaviours in adulthood (Fone and Porkess, 2008; Jones et al., 2011). The behavioural, morphological and neurochemical alterations elicited by post-weaning social isolation (pwSI) have translational significance to several core features that seem to share the neurobiology implicated in the pathophysiology of schizophrenia. Classical and already validated neurochemical alterations include both hyper and hypofunctional dopaminergic neurotransmission in the mesolimbic and mesocortical pathways, respectively, in agreement with the dopaminergic hypothesis suggested for schizophrenia (Howes et al., 2015). On the behavioural level, isolated rats present impaired sensorimotor gating, cognitive deficits, increased aggressive behaviour, reduced social interaction and hyperlocomotion. Remarkably, hyperlocomotion was described before as a suitable marker to confirm the development of the “isolation-induced stress syndrome” before performing more complex behavioural analyses, due to consistent replicability across studies (Fone and Porkess, 2008).

Several lines of evidence suggest impaired prefrontal cortex-hippocampus connectivity as the neuroanatomical substrate involved in spontaneous hyperlocomotion in isolated reared rats. The prefrontal cortex and the hippocampus are critical brain areas of dysfunction in clinical schizophrenia (Glantz and Lewis, 2000; Lewis et al., 2003; Pantelis et al., 2005; Vita and de Peri, 2007), and lack of social stimulation would contribute to reduced volume, besides impaired neurogenesis, plasticity and connectivity in these two brain areas (Lu et al., 2003; Silva-Gómez et al., 2003; Harte et al., 2004; Day-Wilson et al., 2006; Pascual et al., 2007; Ibi et al., 2008; Schubert et al., 2009; Quan et al., 2010; Pereda-Pérez et al., 2013; Biro et al., 2017).

The immune system profoundly affects brain development and function, with cytokines participating in both neurodevelopment and neurogenesis processes (Borsini et al., 2015). Even though the relationship between early-life stress and schizophrenia is widely described (Schafer and Fisher, 2011; Varese et al., 2012), the biological mechanisms underlying this association remain to be elucidated. A current working hypothesis is that life stressors during critical periods of the neurodevelopment trigger an immune dysfunction characterised by abnormal production of inflammatory cytokines (Monji et al., 2013; Knuesel et al., 2014; Baumeister et al., 2015). An imbalance between inflammatory cytokines during early brain development results in abnormal neurotransmission in the central nervous system, affecting brain functioning (Meyer et al., 2011; Khandaker and Dantzer, 2015; Corsi-Zuelli et al., 2017), and therefore, can increase the risk of psychiatric disorders in adulthood, including schizophrenia (Meyer, 2011, 2014; Knuesel et al., 2014).

Crescent interest has been given upon enhanced pro-inflammatory cytokines in the pathophysiology of schizophrenia. In clinical trials, abnormal production of IL-6 and TNF-α in the peripheral blood of patients with schizophrenia and other psychotic disorders is the most replicated finding (Potvin et al., 2008; Miller et al., 2011; Upthegrove et al., 2014; Goldsmith et al., 2016), whereas the participation of anti-inflammatory cytokines, such as IL-10, remains poorly explored (Potvin et al., 2008; Miller et al., 2011; Goldsmith et al., 2016). In the central nervous system, however, besides a pro-inflammatory state, there seem to exist a blunted anti-inflammatory activity, as demonstrated by reduced IL-10 levels in the cortex (Pandey et al., 2017). Nevertheless, it remains unclear whether this blunted anti-inflammatory activity is being translated into the blood.

Despite the pro-inflammatory state reported in schizophrenia, not all patients present with immune dysregulation (Goldsmith et al., 2016). In fact, data on early-stress and inflammation in clinical trials are scarce but provide with clearly heterogenous results (Coelho et al., 2014) that could be influenced by pharmacological treatment, substance abuse and other social and environmental factors. Rodent models provide the opportunity to investigate the inflammatory cytokines simultaneously in the periphery and in the brain in the absence of such influences. In the context of pwSI, however, the few existing studies have focused mainly on peripheral changes of pro-inflammatory cytokines (Möller et al., 2013; Ko and Liu, 2015, 2016; Wang et al., 2017). From these, only one attempted to determine a concomitant change of cytokines (IL-6, IL-1β, and TNF-α) at both the peripheral blood and the hippocampus, indicating a pro-inflammatory state (Wang et al., 2017); nevertheless, this study did not include the measurement of anti-inflammatory cytokines. Conversely, a recently published study found reduced IL-6 and IL-10, but unchanged TNF-α levels in the hippocampus of animals submitted to the pwSI model (Dunphy et al., 2017). However, in this study neither blood cytokines nor cytokines gene expressions were not investigated. To this end, whether the reduced ability to produce anti-inflammatory cytokines is reflected in the peripheral blood remains to be explored, as no existing study included the measurement of this type of cytokine concomitantly at blood and brain in the pwSI model. The existing synergism in blood-brain cytokines would contribute as potential blood biomarkers in psychosis, at least in a subgroup of patients exposed to early trauma.

Given the aforementioned, we evaluated a possible imbalance between protein and gene expression of pro- and anti-inflammatory cytokines (IL-6, TNF-α, and IL-10) simultaneously at both blood and brain tissues (prefrontal cortex and hippocampus) of rats submitted to the pwSI model. We hypothesised that social-isolated animals would present imbalanced levels of both IL-6 and TNF-α together with reduced IL-10 in the brain, and that this deregulation of inflammatory molecules would be reflected in the peripheral blood as well. Additionally, since locomotor habituation depends on a functional prefrontal cortex-hippocampus connectivity (Silva-Gómez et al., 2003), and given the participation of inflammatory cytokines in the neurodevelopment and neurogenesis of these brain sites (Borsini et al., 2015), we have also hypothesised that the aberrant cytokine production would correlate with hyperlocomotion in social isolated-animals.

## Materials and Methods

### Animals

Male *Wistar* rat pups (University of São Paulo, Ribeirão Preto *Campus*) were kept with their lactating dams until weaning. Right after weaning on postnatal day 21, the pups (40 g) were separated from their mothers and randomly divided into two different rearing conditions for a period of 10 weeks: (A) group-housed (*n* = 10; 3–4 per cage); (B) isolation-reared (*n* = 10; housed individually). Handling was performed for cleaning purposes only and by suspending the rats by the tail (5 s) to allocate them into a clean cage. The same person performed all the handling procedure. Rats (grouped or isolated) were housed in the same animal facility room, kept in plastic cages (48.5 × 25.8 × 15.6 cm), in a temperature-controlled room (23 ± 1°C), under 12 h light/dark cycle (lights on 06:30 a.m.) with free access to food and water. Isolated rats were always prevented from any form of contact with a conspecific, although rats could see, hear and smell their mates. The experiments were carried out according to the Brazilian COBEA (Colégio Brasileiro de Experimentação Animal) guidelines, which complies the National Institutes of Health guide for care and use of Laboratory animals (NIH Publications No. 8023, revised 1978). This study was approved by the Ribeirão Preto School of Medicine local ethics committee (024/2016).

### Open Field Test

On postnatal day 91, rats were tested in the open field. The apparatus consisted of a square arena (dimensions 40 × 72 × 72 cm), with the ground separated into 16 equal squares by black lines. All testing was performed in the light phase of the circadian cycle. The number of square crossings of each rat was videotaped for 20 min. Square crossings were evaluated at both the periphery and centre of the arena over the 20 min period divided into four time bins break (0–5; 5–10; 10–15; 15–20 min), using the EthoLog 2.2 software (Ottoni, 2000).

### Sample Processing

A week after the behavioural test, rats were decapitated using isoflurane as a pre-anaesthetic. Trunk blood was collected in 4 mL EDTA-containing tubes, which were immediately stored on ice for plasma and leucocytes preparation. For plasma collection, the tubes were centrifuged at 3,500 rpm at 4°C for 10 min. Peripheral blood leucocytes were collected right after the blood collection, by low-density gradient centrifugation via Ficoll-Paque PLUS (GE Healthcare), as previously described (Do Prado et al., 2017). All the samples were stored at -80°C until the day of the assay procedure.

The whole brains were extracted and the both hemispheres of regions of interest (prefrontal cortex and hippocampus) were manually dissected, frozen in isopentane and stored at -80°C before use. We chose randomly which hemisphere (left, right) would be used per animal, so we could guarantee that both groups (isolated and grouped) contained the right and left hemispheres for both the multiplex and gene expression assays. For cytokines quantification, the brain tissues were weighted, homogenised in lysis buffer solution with a protease inhibitor cocktail (Sigma-Aldrich, St. Louis, MO, United States) using an ultrasonic dismembrator (model 100; Fisher Scientific) at 4°C operating three rapid pulses (<1 s each), centrifuged (3,500 rpm at 4°C for 10 min), and the supernatant stored at -80°C until ready for cytokine measurement.

### Multiplex Assay

Cytokine measurement (IL-6, TNF-α, and IL-10) was performed in plasma and in tissue supernatant of brain areas collected (prefrontal cortex, hippocampus) from both social and isolated-reared rats. The three cytokines were simultaneously quantified from a single sample of each animal (25 μL) using the Milliplex MAP Rat Cytokine/Chemokine magnetic bead panel (#RECYTMAG-65K; EDM Millipore, Billerica, MA, United States^1^). The assay was performed in 96-well plates according to the manufacturer’s instructions and the results were expressed in pg/mL. Briefly, each assay plate layout consisted of seven standards, two positive controls, two blank wells, all runned in duplicate, and up to 76 samples, as previously described (Lu et al., 2017). Results were analysed on a Luminex-200 System (Luminex, Austin, TX, United States) and reported on the xPOTENT software version 3.1.

Sample wells with bead count <50 were excluded from the analysis, according to manufacturer’s instructions. Cytokines concentrations were calculated through the five-parameter logistic curve-fitting method using the median fluorescence intensity. All data were corrected using the Milliplex Analyst software.

### Gene Expression Analysis

Gene expression analyses were conducted for *IL-6*, *TNF*, and *IL-10* at prefrontal cortex, hippocampus, and peripheral leucocytes. Total RNA was extracted using the All prep DNA/RNA mini kit (Qiagen, Valencia). The tissue (flash-frozen) was disrupted using syringe and needle in Buffer RLT Plus (lysis buffer containing β-ME). The kit guarantee efficient purification of high-quality DNA and RNA without the need for additional RNase and DNase digestions. Biological samples are first lysed and homogenised in a highly denaturing guanidine-isothiocyanate–containing buffer, which immediately inactivates DNases and RNases to ensure isolation of intact DNA and RNA. The lysate is then passed through an AllPrep DNA spin column. This column, in combination with the high-salt buffer, allows selective and efficient binding of genomic DNA. The column is washed and pure, ready-to-use DNA is then eluted.

Ethanol is added to the flow-through from the AllPrep DNA spin column to provide appropriate binding conditions for RNA, and the sample is then applied to an RNeasy spin column, where total RNA binds to the membrane and contaminants are efficiently washed away. High-quality RNA is then eluted in 30 μl of water.

NanoDrop^®^ ND-1000 spectrophotometer (Nanodrop, Wilmington, DE, United States) was used to determine the purity and quantity of the RNA samples. cDNA was synthesised using the High-Capacity cDNA Reverse Transcription Kit (Life Technologies, Foster City, CA, United States), by using approximately 400 ng of each RNA sample, and 100 ng were then diluted in H_2_O, mixed with TaqMan^®^ Universal PCR Master Mix (Life Technologies) and disposed in a 96 well plate. The Real-Time quantitative PCR (RT-qPCR) primers and probes were chosen following the best coverage and mispriming absence according to manufacturer’s guarantee (Applied Biosystems^2^, United States). Probes and primers of 3 target genes (*IL-6:* Rn01410330_m1; *TNF:* Rn01525859_g1 and *IL-10:* Rn01483988_g1) and two housekeeping genes (β-actin, *ACTB;* proteasome subunit beta type-2, *PSMB2*) were preloaded in the wells and the experiments were performed in accordance with the manufacturer’s instructions using the ViiATM 7 Real-Time PCR System (Life Technologies). Gene expression was quantified using the Comparative threshold (Ct) method (ΔΔCt Method) (Livak and Schmittgen, 2001; Schmittgen and Livak, 2008), and the amount of target gene was normalised to the housekeeping genes and determined by 2^-ΔΔCt^, as previously described (Julian et al., 2014; Walder et al., 2014), with relative expression levels reported as fold change. Ct values higher than the cut-off of 35 were not considered as a reliable expression value, according to manufacturer’s recommendations (Life Technologies Corporation, 2011), and therefore were excluded from the statistical analysis.

### Data Analysis

Statistical analysis was performed using the SPSS, version 24.0 (IBM Corp: Armonk, NY, United States) and R statistical software (Package corrplot), version 3.5.1 (R Core Team, 2018). Behavioural data were analysed with repeated measures ANOVA (*Hotelling’s Trace*) considering the experimental condition (grouped or isolated) as the between factor, and the number of crossings at the arena (periphery or centre) and time (0–5; 5–10; 10–15; 15–20 min) as within factors. Bonferroni was set as the *post hoc* test. Cytokines measurements were logarithmic transformed for the statistical analysis with the purpose of normalising the distribution errors and homogenising variances, but figures are expressed as raw values. Significant differences between groups were determined by the Student’s *t*-test. *Spearman rho* correlation matrix was used to explore significant correlations among the variables cytokines (protein and mRNA) in blood and brain, as well as cytokines and total number of square crossings in the open field for isolated-reared animals. Values of *p* ≤ 0.05 were considered significant.

## Results

### Behavioural Data

**FIGURE 1:**
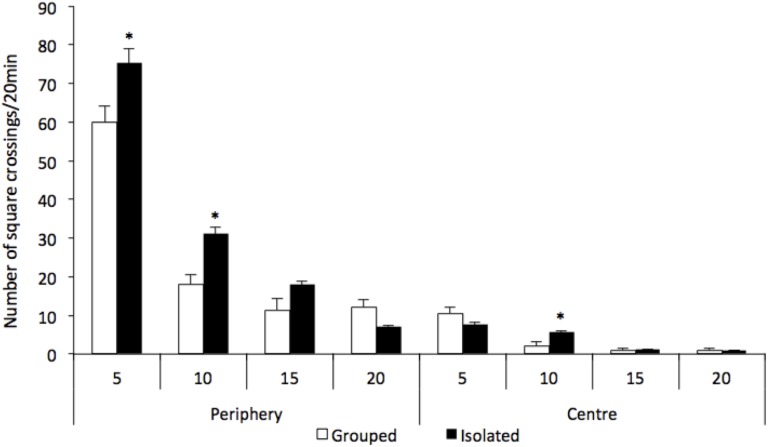
Effect of rearing condition (isolated vs. grouped) at spontaneous number of crossings at the open field (20 min). Number of crossings is represented as 5-min time bins over the 20 min of test. Repeated measures ANOVA showed a significant group and local and time interaction and *post hoc* analysis revealed that rats reared isolated (*n* = 8) presented hyperlocomotion at the periphery of the arena at 5 and 10-min time bins, and at the centre of the arena at 10-min time bin when compared to grouped rats (*n* = 9). Data are expressed as mean ± S.E.M. ^∗^*p* < 0.05 compared to grouped rats.

There were significant differences between groups regarding the locomotion in the open field test [group factor *F*_(1,15)_ = 419.191; *p* = 0.030; group and local and time interaction *F*_(3,13)_ = 7.047; *p* = 0.005] (Figure 1). Isolated-reared rats habituated slowly, as the number of square crossings was significantly greater for this group when compared to controls [local factor *F*_(1,15)_ = 294.402; *p* < 0.001]. Rats reared isolated presented hyperlocomotion at the two first time bins (0–5 and 5–10 min) at periphery of the arena when compared to grouped [0–5 min: *F*_(1,15)_ = 6.209; *p* = 0.025; 5–10 min: *F*_(1,15)_ = 14.272; *p* = 0.002, respectively]. A higher number of crossings during the second time bin (5–10 min) at the centre of the arena was also detected in isolated reared animals when compared to grouped [5–10 min: *F*_(1,15)_ = 6.452, *p* = 0.023].

### Plasma Cytokines Concentrations

**FIGURE 2:**
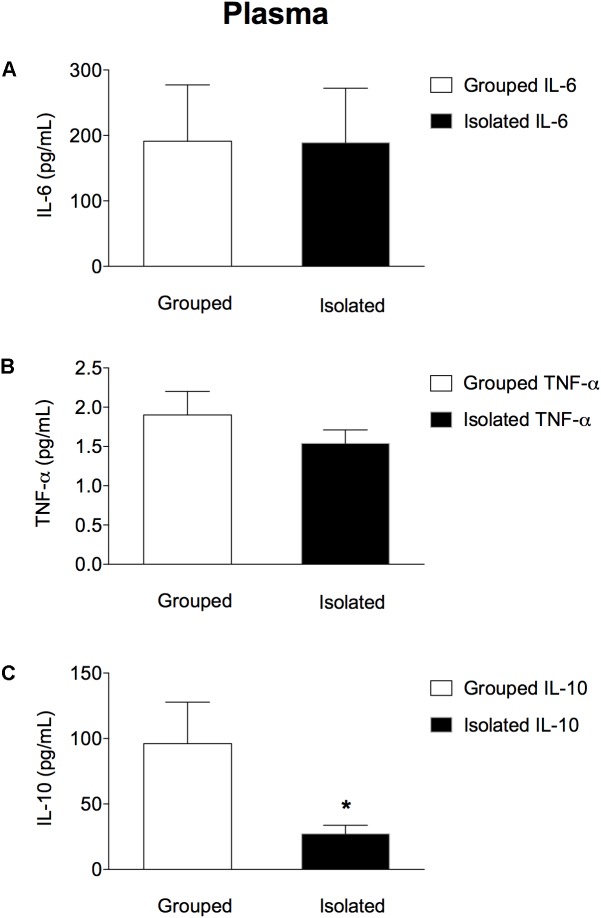
Effect of rearing condition (isolated vs. grouped) on cytokines plasma levels of rats exposed to 10 weeks of social isolation. No difference was found for **(A)** IL-6 or **(B)** TNF-α plasma concentration in isolated rats (*n* = 10) when compared to grouped housed (*n* = 9). However, isolated rats (*n* = 10) showed lower **(C)** IL-10 plasma concentration when compared to grouped-housed (*n* = 9). Data are expressed as mean ± S.E.M and given as pg/mL. ^∗^*p* < 0.05 (Student’s *t*-test) compared to grouped rats.

In the peripheral blood (Figures 2A–C) isolated-reared rats had lower c) IL-10 plasma levels when compared to grouped-housed animals (*t* = 2.442; d.f. = 17; *p* = 0.025), whereas no significant differences between the two groups were found either for a) IL-6 (*t* = 0.400; d.f. = 17; *p* = 0.694) or b) TNF-α (*t* = 1.065; d.f. = 17; *p* = 0.302).

### Brain Cytokines Concentrations

**FIGURE 3:**
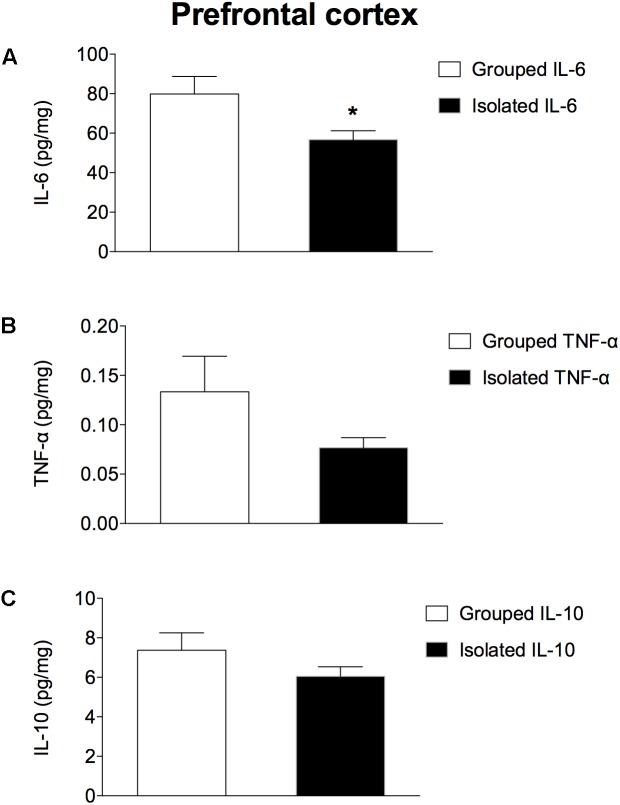
Effect of rearing condition (isolated vs. grouped) on cytokines in the prefrontal cortex of rats exposed to 10 weeks of social isolation. Isolated rats (*n* = 9) had reduced **(A)** IL-6, but no difference was found for **(B)** TNF-α or **(C)** IL-10 in comparison to grouped rats (*n* = 9). Data are expressed as mean ± S.E.M and given as pg/mg. ^∗^*p* < 0.05 (Student’s *t*-test) compared to grouped rats.

**FIGURE 4:**
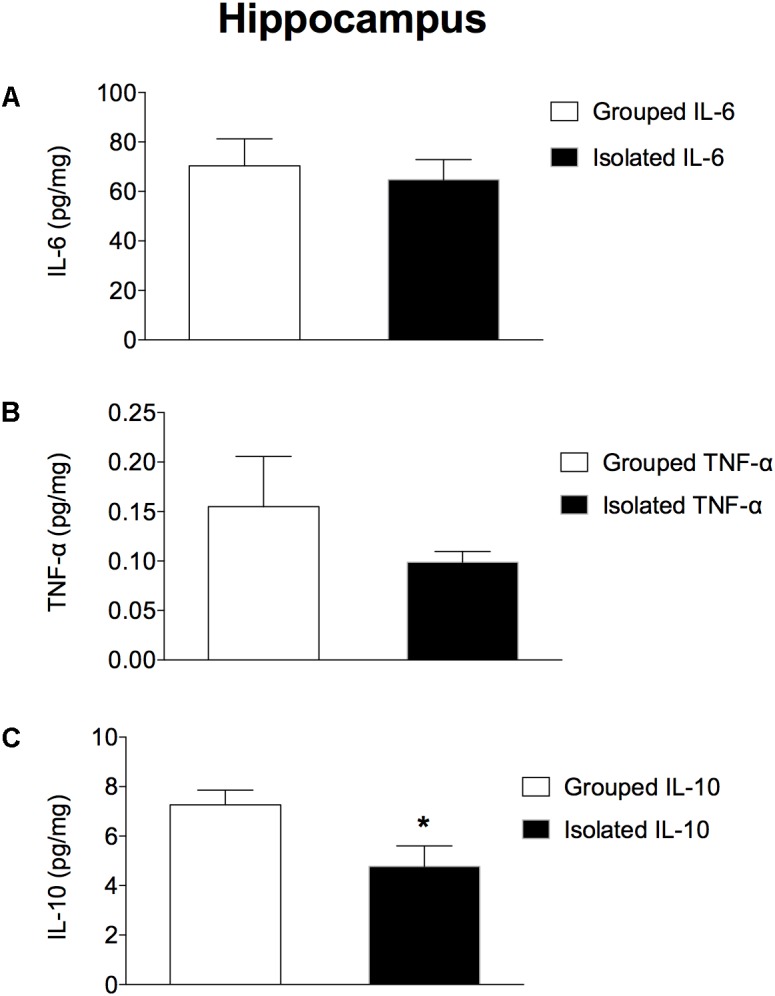
Effect of rearing condition (isolated vs. grouped) on cytokines in the hippocampus of rats exposed to 10 weeks of social isolation. Rats reared isolated (*n* = 9) presented lower levels of **(C)** IL-10, with no differences for **(B)** TNF-α or **(A)** IL-6 when compared to controls (*n* = 8). Data are expressed as mean ± S.E.M and given as pg/mg. ^∗^*p* < 0.05 (Student’s *t*-test) compared to grouped rats.

In the prefrontal cortex (Figures 3A–C), isolated-reared rats had reduced levels of a) IL-6 (*t* = 2.281; d.f. = 16; *p* = 0.037), but not of b) TNF-α (*t* = 1.523; d.f. = 16; *p* = 0.147) or c) IL-10 (*t* = 1.073; d.f. = 16; *p* = 0.299) when compared to controls. In the hippocampus (Figures 4A–C), the concentration of c) IL-10 was significantly lower than in group-housed rats (*t* = 2.347; d.f. = 15; *p* = 0.032). Conversely, no difference was found for a) IL-6 (*t* = 0.226; d.f. = 15; *p* = 0.824) or b) TNF-α (*t* = 0.114; d.f. = 15; *p* = 0.323).

### Cytokines Gene Expression

**FIGURE 5:**
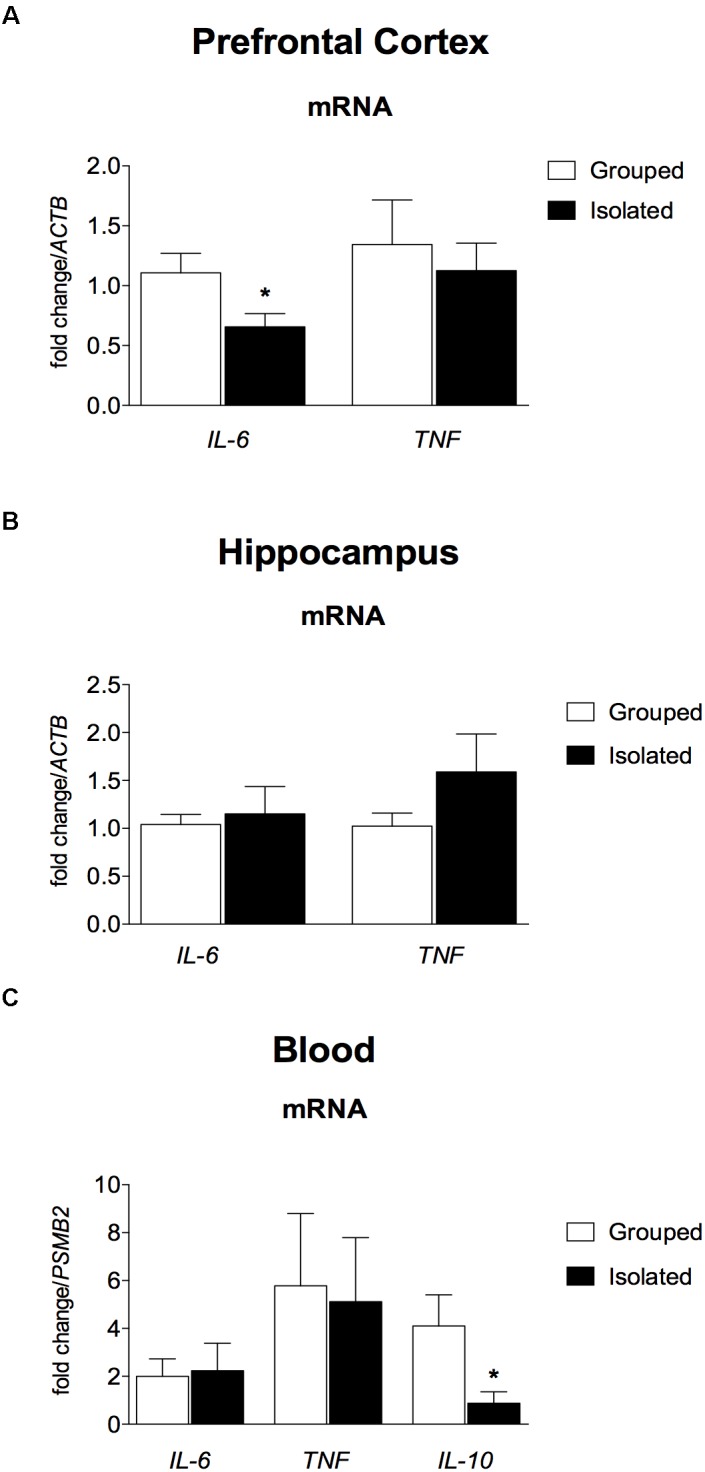
Effect of rearing condition (isolated vs. grouped) on cytokines gene expression in the pre-frontal cortex, hippocampus, and peripheral blood of rats after 10 weeks of social isolation. **(A)** Prefrontal Cortex: Isolated-reared rats (*n* = 9) presented reduced *IL-6* mRNA in the prefrontal cortex when compared to controls (*n* = 10). **(B)** Hippocampus: No difference was found for isolated (*n* = 9) or grouped-reared (*n* = 10) rats in *IL-6* or *TNF* mRNA. **(C)** Peripheral blood: Isolated-reared rats (*n* = 7) had reduced IL-10 mRNA in peripheral blood leucocytes than controls (*n* = 7). Cytokine mRNA expression was measured by qRT-PCR. Data are expressed as mean fold change ± S.E.M in mRNA levels using the housekeeping genes (*ACTB, PSMB2*) as reference. ^∗^*p* < 0.05 (Student’s *t*-test) compared to grouped rats.

The RT-qPCR showed that *ACTB* and *PSMB2* were expressed at a stable level across all the samples. In the brain (Figures 5A,B), Ct values were higher than the cut-off of 35 for *IL-10*, which are considered below the detection limit of expression (data not shown). In the prefrontal cortex, isolated rats presented down regulated *IL-6* mRNA expression (*t* = 2.234; d.f. = 17; *p* = 0.039; Figure 5A, *left*), but not in the hippocampus (*t* = 0.383; d.f. = 17; *p* = 0.707; Figure 5B, *left*). No difference was found for *TNF* gene expression (prefrontal cortex: *t* = 0.485; d.f. = 17; *p* = 0.634; Figure 5A, *right*; hippocampus: *t* = 1.407; d.f. = 17; *p* = 0.177; Figure 5B, *right*). In the peripheral blood (Figure 5C) *IL-10* mRNA was significantly down regulated in isolated-reared rats (*t* = 2.342; d.f. = 12; *p* = 0.049), with no significant differences in IL-6 (*t* = 0.172; d.f. = 12; *p* = 0.867) or TNF mRNA (*t* = 0.166; d.f. = 12; *p* = 0.871).

### Behaviour, Blood and Brain Cytokines Correlations

**FIGURE 6:**
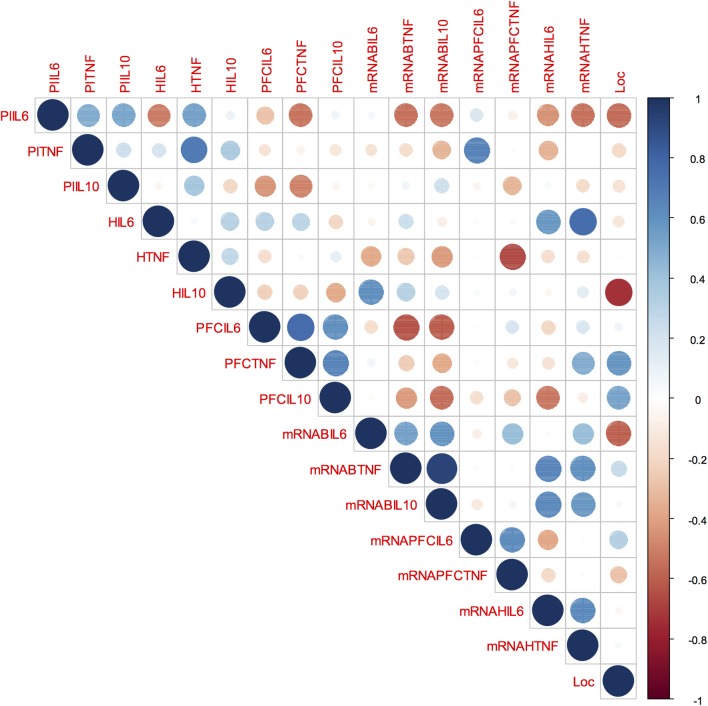
Behaviour, blood and brain cytokines correlation in isolated-reared rats: Correlation matrix displaying the positive (blue) and negative (red) correlations between behaviour, blood, and brain cytokines data. Colour depth and size variation of circles in the figure indicate the correlation strength. Number range from –1 to 1 represent Spearman’s rank correlation coefficients of variables on horizontal and vertical axes. PIL6, plasma IL-6; PlTNF, plasma TNF; PlIL-10, plasma IL-10; HIL6, IL-6 levels in the hippocampus; HTNF, TNF levels in the hippocampus; HIL10, IL10 levels in the hippocampus; PFCIL6, IL-6 levels in the prefrontal cortex; PFCTNF, TNF levels in the prefrontal cortex; PFCIL10, IL-10 levels in the prefrontal cortex; mRNABIL6, IL-6 mRNA expression in the peripheral blood; mRNABTNF, TNF mRNA expression in the peripheral blood; mRNABIL10, IL-10 mRNA expression in the peripheral blood; mRNAPFCIL6, IL-6 mRNA expression in the prefrontal cortex; mRNAPFCTNF, TNF mRNA expression in the prefrontal cortex; mRNAHIL6, IL-6 mRNA expression in the hippocampus; mRNAHTNF, TNF mRNA expression in the hippocampus; Loc, locomotor activity in the open field.

Our correlation matrix (Figure 6) shows that isolated-reared rats presented a significant negative correlation between the total number of square crossings and IL-10 concentration in the hippocampus (rho: -0.739; *p* = 0.029). No other significant correlations were found between locomotor behaviour and the remaining cytokines in isolated-reared rats (*p* > 0.05 for all).

Plasma TNF-α correlated positively with TNF-α protein in the hippocampus (rho: 0.69; *p* = 0.039) and with IL-6 mRNA in the prefrontal cortex (rho: 0.66; *p* = 0.049), whereas blood *TNF* mRNA correlated positively with blood *IL-10* mRNA (rho: 0.92; *p* = 0.002). In the prefrontal cortex, TNF-α protein correlated positively with both IL-6 (0.76; *p* = 0.016) and IL-10 protein (rho: 0.66; *p* = 0.049). In the hippocampus, *TNF* mRNA correlated positively with IL-6 protein (0.75; *p* = 0.030) but hippocampal TNF protein correlated negatively with TNF mRNA in the prefrontal cortex (rho: -0.66; *p* = 0.049).

## Discussion

The present study demonstrates, for the first time, that rats exposed to prolonged periods of social isolation since weaning have a set of peripheral and central inflammatory changes in adulthood, characterised by reduced IL-10 protein and gene expression in the blood and reduced IL-10 protein in the hippocampus, along with decreased IL-6 and its mRNA expression in the prefrontal cortex. Besides, we demonstrate that cytokines correlate to one-another in the different compartments analysed and that IL-10 hippocampal concentrations are negatively correlated with locomotion in the open field. Of particular note, these results suggest that prolonged early-life adverse events reduce the anti-inflammatory cytokine IL-10 in blood and brain, and that the latter may contribute to the occurrence of abnormal behaviour in adulthood.

The pattern of spontaneous hyperlocomotion observed in our study is consistent with other investigations submitting *Wistar* rats to the same pwSI protocol (Domeney and Feldon, 1998; Heidbreder et al., 2000). Spontaneous hyperlocomotion is described as one of the earliest (appearing after 2 weeks of social isolation) and one of the most robust observation in isolated-reared rodents, and reflects an inability to habituate following placement in a novel environment (Fone and Porkess, 2008). Accordingly, in the present study, we confirm the robustness and existence of hyperlocomotion even after 10 weeks of social isolation.

Increased pro-inflammatory cytokines plasma concentration in chronic isolated rats was not confirmed in this study; instead, we observed reduced peripheral protein and gene expression of the anti-inflammatory cytokine IL-10, in the absence of any concomitant changes regarding IL-6 or TNF-α in isolated reared rats. From the existing studies investigating peripheral blood cytokines in rodents under pwSI, data are still conflicting, with some pointing to enhanced (Möller et al., 2013; Ko and Liu, 2015, 2016; Wang et al., 2017), decreased or unchanged levels (Möller et al., 2013; Dunphy et al., 2017), especially in IL-1β, IL-6, TNF-α, and INF-γ, whereas changes in IL-10 are absent (Ko and Liu, 2015) or not investigated (Möller et al., 2013; Wang et al., 2017). Parallel to the observed reduced protein and gene expression of IL-10 in the peripheral blood, we have also observed the same direction of change of this cytokine in the hippocampus of isolated reared animals, again in the absence of modifications on the other two other cytokines investigated.

Congruent to our finds in the peripheral blood not showing enhanced pro-inflammatory cytokines, we also observed reduced IL-6 protein and gene expression in the prefrontal cortex of these animals. Although our results differ from a previous study, which detected enhanced IL-1β, IL-6, and TNF-α in the hippocampus of rats under social isolation (Wang et al., 2017), we are in agreement with a recent study reporting decreased levels of both IL-6 and IL-10 in the brain of isolated-reared rats (Dunphy et al., 2017), in the absence of abnormalities of other pro-inflammatory markers.

These discrepancies could be due to methodological factors, including those related to the rat strain used, and probably more important, the length of social isolation period applied, as both can account for behavioural and inflammatory response discrepancies. Decreased inflammatory cytokines have been reported in chronic long-term social isolation protocols, consisting of 8–10 weeks of social isolation (Möller et al., 2013; Cruces et al., 2014; Dunphy et al., 2017), while the enhanced pro-inflammatory cytokines have been observed in animals exposed to a shorter social isolation period (4–6 weeks) (Krügel et al., 2014; Ko and Liu, 2015; Wang et al., 2017).

Indeed, clinical studies of psychosis also support that changes in inflammatory markers may be variable. For instance, in the *postmortem* schizophrenia brain, pro-inflammatory cytokines concentrations seem to be inconsistent across studies, with some showing enhanced or decreased concentrations (Trépanier et al., 2016; van Kesteren et al., 2017), but the scarce data regarding anti-inflammatory factors point to reduced concentrations of the soluble IL-2 receptor in the cerebrospinal fluid (Wang and Miller, 2017), and reduced levels of both IL-1 receptor antagonist (IL-1RA) (van Kesteren et al., 2017) and IL-10 in the cortex (Pandey et al., 2017). *In vivo* studies using PET scan are in agreement, showing increased glia activation in the central nervous system of patients in the early stages (van Berckel et al., 2008), but not in chronic stages of psychosis (Takano et al., 2010; Kenk et al., 2015). Besides that, systematic reviews of clinical studies on early-life adversities and inflammation highlight that different types and duration of early social adversities may promote changes in the inflammatory system in many diverse directions (Coelho et al., 2014).

It is not clear yet whether cytokines in the peripheral blood accompany cytokines changes in the central nervous system. Different from the previous studies, we were able to test correlations between pro- and anti-inflammatory cytokines in blood and brain areas of rats exposed to pwSI. Our correlation matrix suggests that cytokines tend to correlate to one-another in the two different brain regions analysed, the majority of them revealing positive correlations, except for *TNF mRNA* in the prefrontal cortex, which was negatively correlated with TNF-α protein in the hippocampus. Cytokines tend to have a dynamic response, presenting with a complex positive and negative feedback loop to regulate one-another expression, and dissociation between gene and protein expression are not uncommon, with some hypothesis suggesting biological factors related to transcriptional, translational or post-translational changes (Maier et al., 2009).

Although IL-10 protein and gene expression in the peripheral blood were not statistically correlated with IL-10 in the brain, isolated-reared rats presented reduced levels of this cytokine in both compartments, and hippocampal IL-10 correlated negatively with locomotion in the open field. The reasons for such dissociations are unclear but the existence of distinct regulatory mechanisms in the brain and blood that could contribute to the observed discrepancies have been suggested (Wang and Miller, 2017). For instance, some studies point that cytokine expression in brain and blood typically occurs independently and in an age and region-specific manner, suggesting an independent role of cytokines in blood and brain (Garay et al., 2013).

In fact, IL-10 is a potent anti-inflammatory cytokine, produced mainly by M-2 type macrophages, Th-2 lymphocytes, T-regulatory lymphocytes and B regulatory cells. In the central nervous system, IL-10 is also produced by microglia, and astrocytes (Ledeboer et al., 2002; Roque et al., 2009; Pérez-de Puig et al., 2013). There may be several pathways through which IL-10 influences behaviour. One indirect mechanism may have to do with its anti-inflammatory properties in dampen an aberrant production of pro-inflammatory cytokines. A more direct mechanism, however, involves its role in the central nervous system. IL-10 protects glial cells against lipopolysaccharide (LPS)/IFN-γ induced cytotoxicity (Molina-Holgado et al., 2001), and prevents glutamate-mediated neuronal death by blocking proapoptotic proteins and by enhancing anti-apoptotic factors (Bachis et al., 2001). IL-10 also regulates neurogenesis processes, and polarises macrophages and microglia cells towards a protective state (Strle et al., 2001; Ledeboer et al., 2002; Couper et al., 2008; Roque et al., 2009; Kwilasz et al., 2015; Lobo-Silva et al., 2016). The maternal immune activation model of psychosis leads to decreased IL-10 levels at both the maternal fluids and in the foetal brain, causing a set of psychotic-like behaviour disturbances, which are all prevented by IL-10 overexpression in the macrophages of pregnant dams (Smith et al., 2007; Oskvig et al., 2012; Meyer, 2014). In psychosis, reduced IL-10 levels were negatively correlated with negative and cognitive symptoms (Xiu et al., 2014).

The primary strength of our study is the investigation of blood and brain pro- and anti-inflammatory cytokines in a non-pharmacological animal model of schizophrenia. Our results bring the question to whether peripheral blood cytokines may reflect cytokines in the central nervous system and suggest that the degree of such correlation is far from perfect. We also suggest that chronic stress may reduce anti-inflammatory cytokine in blood and brain. Despite it deserves more exploration, this synergism would open the opportunity to target potential blood biomarkers to identify subgroup of patients exposed to early trauma. Given the fact that pwSI represents a pure environmental and non-pharmacological pre-clinical model of schizophrenia with the appropriate triad of validity (Fone and Porkess, 2008), this study emphasises the participation of early-traumatic environmental factors in the inflammatory hypothesis of schizophrenia.

A limitation of this study is the fact that the behavioural investigation was focused only on hyperlocomotion. However, there is evidence showing that a combination of multiple stressors influence cytokine response (Lovelock and Deak, 2017), and consequently could mask the effect of the prolonged chronic social isolation on cytokine production. We selected hyperlocomotion because it is considered the most consistent response to social isolation, representing a suitable marker to confirm the development of the “*isolation-induced stress syndrome*” before performing more complex behavioural analyses (Fone and Porkess, 2008). Another limitation is that we were unable to include the measurement of other cytokines; therefore the investigation of other inflammatory markers would deserve further exploration in the pwSI model. However, in order to minimise possible cytokine quantification errors, we used Luminex x-map technology for cytokine measurement, which provides the most consistent sensitivity for cytokines concentration using a minimal sample volume (25 μL), enabling patterns of numerous cytokines to be examined, an approach that is not possible with conventional technologies (Belzeaux et al., 2017). Besides, we employed TaqMan technology for RT-qPCR, which leads to less non-specific products and more reliable results (Yin et al., 2001), different from studies using SYBR Green.

## Conclusion

Taken together, our study shows for the first time that exposing rats to prolonged periods of social isolation since weaning reduces the protein and gene expression of the anti-inflammatory cytokine IL-10, and this reduction is translated from blood-to-brain. Interestingly, reduced IL-10 in the hippocampus correlated negatively with locomotor activity in the open field. Our results also showed that cytokines tend to correlate to one-another among the compartments investigated, although blood and brain correlations could be questionable. We emphasise the need of more investigations focused on the association between early trauma and inflammation in schizophrenia, with attention to the type and duration of trauma, as well as more investigations aiming to correlate blood and brain cytokines, as this synergism would open the opportunity to identify subgroup of patients exposed to early trauma.

## Author Contributions

FC-Z designed the study, conducted the experimental procedures, analysed and interpreted the data, and wrote the first draft of the manuscript. HF designed the study, obtained the approval from the ethical committee, wrote the protocol, was in charge of the data collection, including molecular experiments, and revised the manuscript. CL collected data and collaborated on the statistical analysis. RS helped on behavioural data analysis. GB collaborated with the protocol design and supervised cytokines measurements. SJ collaborated in the design of the study and supervised data collection. PM obtained funding and critically revised the manuscript. PL-J obtained funding, collaborated with data analyses and with the interpretation of the results, and critically revised the manuscript. CD-B conceived the study, obtained funding, obtained the approval from the ethical committee, supervised data analyses, participated in the interpretation of the results, and critically revised the manuscript. All the authors approved the final version of the manuscript.

## Conflict of Interest Statement

The authors declare that the research was conducted in the absence of any commercial or financial relationships that could be construed as a potential conflict of interest.

## Footnotes

1 https://www.emdmillipore.com/US/en

2 http://www.thermofisher.com/br/en/home/life-science/pcr/real-time-pcr/real-time-pcr-assays.html
